# FlowFP: A Bioconductor Package for Fingerprinting Flow Cytometric Data

**DOI:** 10.1155/2009/193947

**Published:** 2009-09-24

**Authors:** Wade T. Rogers, Herbert A. Holyst

**Affiliations:** Department of Pathology and Laboratory Medicine, School of Medicine, University of Pennsylvania, 207 John Morgan Bldg., Philadelphia, PA 19104-6082, USA

## Abstract

A new software package called flowFP for the analysis of flow cytometry data is introduced. The package, which is tightly integrated with other Bioconductor software for analysis of flow cytometry, provides tools to transform raw flow cytometry data into a form suitable for direct input into conventional statistical analysis and empirical modeling software tools. The approach of flowFP is to generate a description of the multivariate probability distribution function of flow cytometry data in the form of a “fingerprint.” As such, it is independent of a presumptive functional form for the distribution, in contrast with model-based methods such as Gaussian Mixture Modeling. FlowFP is computationally efficient and able to handle extremely large flow cytometry data sets of arbitrary dimensionality. Algorithms and software implementation of the package are described. Use of the software is exemplified with applications to data quality control and to the automated classification of Acute Myeloid Leukemia.

## 1. Introduction

Flow cytometry (FC) produces multidimensional biological information at the level of the cellular compartment, and over very large numbers of cells. As such it is ideally suited to a wide variety of investigations for which cellular context and large sample observations are important. In recent years the technology of FC has undergone appreciable development [1, 2] with the introduction of digital signal processing electronics [3], multiple lasers, increasing numbers of fluorescence detectors, and robotic automation, both in sample preparation [4] and in instrumental data collection [5]. The recent development of new reagents [6] that enable increasing assay complexity has also been rapid and accelerating. Given the scope and pace of these developments, the bottleneck in many FC experiments has shifted from the wet laboratory to the computer laboratory; that is to say, data analysis [1].

FC data are typically analyzed using graphically driven approaches. Subsets of cells (events) are delineated usually in one- or two-dimensional histograms or “dotplots” in a procedure termed “gating.” Gates of differing shapes including rectangular, circular, elliptical, or arbitrary polygonal contours may be specified. The gating process is frequently applied in a sequential fashion, with the numbers of events inside successive gates falling monotonically from step to step. Subsets determined via gating are typically then quantified with respect to their expression patterns in the dimensions of multiparameter space not utilized for gating, often by simply counting proportions of the subsets that are positive or negative for each of the markers of interest for that subset. Several commercially available software packages have been extensively optimized to support this kind of visually guided analysis workflow, for example, FlowJo (Treestar Inc, Ashland, OR), WinList (Verity Software House, Topsham, ME), and FCSExpress (De Novo Software, Los Angeles, CA).

Manual gating is a highly effective means of analysis of flow cytometry data, especially in cases where the application of expert judgment in the visual design of gating strategies may be able to isolate events of biological interest in the presence of confounding experimental (or biological) variations that will be difficult to account for automatically. Nevertheless, manual gating has three main drawbacks [7–9]. First, the choice of gates is often subjective, particularly in the not-unusual situation where the distribution is broad and smooth. This lack of objective criteria is problematic, especially when different samples may show different types of “excursions” from the average/normal case. Second, because gates are specified by manually drawing regions on a graph using a computer mouse, the process is very labor intensive and time consuming. Finally, because gating and regions of interest are determined by the data analyst based on his or her experience, there may be interesting and informative features that exist within the full ungated multivariate distribution of events but that nevertheless escape detection in this analysis paradigm.

A number of automated gating procedures have been developed with the aim of reducing tedium as well as increasing objectivity in the gating process. Notwithstanding this, a strong need still remains for computational tools that transform and represent multiparameter flow cytometric data in a form efficiently amenable to machine learning and data mining.

We have developed a software package called flowFP to address these limitations in conventional approaches to the analysis of FC data. The broad aim of the package is to directly transform raw FC list-mode data into a representation suitable for direct input to other statistical analysis and empirical modeling tools. Thus, it is useful to think of flowFP as an intermediate step between the acquisition of high-throughput FC data on the one hand, and empirical modeling, machine learning, and knowledge discovery on the other.

## 2. Materials and Methods

### 2.1. Algorithm Description

The software package described herein, flowFP, implements and integrates ideas put forth in [10, 11]. FlowFP utilizes the Probability Binning (PB) algorithm [10] to subdivide multivariate space into hyper-rectangular regions that contain nearly equal numbers of events. According to the vernacular of flow cytometry, the axes describing a multivariate space are referred to as “parameters.” Here we will use the term “variable” so as to avoid confusion with the nomenclature of “parameter” as used in the statistics literature. Regions (bins) are determined by (a) finding the variable whose variance is highest, (b) dividing the population at the median of this variable which results in two bins, each with half of the events, and (c) repeating this process for each subset in turn. Thus, at the first level of binning the population is divided into two bins. At the second level, each of the two “parent” bins is divided into two “daughter” bins, and so forth. The final number of bins *n* is determined by the number of levels *l* of recursive subdivision, such that *n* = 2^*l*^. 

This binning procedure is typically carried out for a collection of samples (instances), called a “training set.” The result of the process models the structure of the multivariate space occupied by the training set by the way it constructs bins of varying size and shape and is thus termed a “model” of the space (not to be confused with modeling approaches that fit data to a parameterized model or set of models). The model is then applied to another set of samples (which may or may not include instances from the training set). This operation results in a feature vector of event counts in each bin of the model for each instance in the set. These feature vectors are, in the context of a specific model, a unique description of the multivariate probability distribution function for each instance in the set of samples, and thus are aptly referred to as “fingerprints.”

Although flowFP generates bins using the PB algorithm, the way it utilizes the resulting fingerprints is similar to the methods described in [11]. Each element of a fingerprint represents the number of events in a particular subregion of the model. Although it may not be known *a priori* which of these regions are informative with respect to an experimental question, it is possible to determine this by using appropriate statistical tests, along with corrections for multiple comparisons, to ascertain which regions (if any) are differentially populated in two or more groups of samples. If we regard the number of events in a bin as one of *n* features describing an instance, then the statistical determination of informative subregions is clearly seen to be a feature selection procedure. 

Fingerprint features are useful in two distinct modes. First, all or a selected subset of features can be used in clustering or classification approaches to predict the class of an instance based on its similarity to groups of instances. Second, the events within selected, highly informative bins can be visualized within their broader multivariate context in order to interpret the output of the modeling process. This step is crucial in that it provides a means to develop new hypotheses for FC-derived biomarkers within the context of existing reagent panels.

### 2.2. Software Implementation

FlowFP is implemented in the open-source R Statistical Computing Environment [12] and is freely available as part of Bioconductor [13]. Within Bioconductor a framework has been created for handling FC data known as flowCore [14, 15]. FlowFP is one of a growing number of Bioconductor packages integrated within this framework and thus able to interoperate with other flowCore-compliant tools as well as with the full range of downstream statistical analysis and machine learning tools available in R. This integration enables flexible creation of powerful high-throughput analysis procedures for large FC data sets.

FlowFP uses the S4 object-oriented facility of R. Computationally intensive parts are written in the C programming language for efficiency. FlowFP is built around a set of three S4 classes, each with a constructor of the same name as the class name. In addition there are a number of methods for data accession, manipulation, and visualization.

#### 2.2.1. FlowFPModel

FlowFPModel is the fundamental class for the flowFP package. The flowFPModel constructor takes a collection of one or more list-mode instances which are represented in the flowCore framework as a flowFrame (for a single instance) or a flowSet (for a collection of instances), respectively (henceforth we will refer to flowFrames and flowSets, the original list-mode data being implied). In addition to the required argument, flowFPModel has optional arguments that allow control over the number of levels of recursive subdivision and the set of variables to be considered in the binning process. By default all variables in the input flowSet are considered, but if this argument is provided, any variables not listed are ignored. The constructor emits an object of type flowFPModel, which encapsulates a complete representation of the binning process that is used later to construct fingerprints.

#### 2.2.2. FlowFP

The flowFP constructor takes a flowFrame or a flowSet as its only required argument, and an optional flowFPModel. If no flowFPModel is supplied, flowFP computes a model (by calling flowFPModel internally). Regardless the source of the model, flowFP applies the model to each of the instances in its input. The resulting flowFP object extends the flowFPModel class and contains two additional important slots to store a matrix of counts and a list of tags. The counts matrix has dimensions *m* × *n*, where *m* is the number of instances in the input flowSet (or one if a flowFrame is provided), and *n* is the number of features in the model. The tags slot is a list of *m* vectors, each of which has *e* elements, where *e* is the number of events in the corresponding frame in the input flowSet. The value for each element of the tag vector represents the bin number into which the corresponding event fell during the fingerprinting procedure. This is useful for visualization or gating based on fingerprints as will be illustrated below.

#### 2.2.3. FlowFPPlex

The flowFPPlex is a container object which facilitates combining, processing, and visualizing large collections of flowFP objects which are all derived from the same set of instances. The flowFPPlex constructor takes a list of flowFP objects. The flowFPPlex manages the logical association of a set of flowFP descriptions. In particular, it extends the counts matrices of its members “horizontally” so as to create a unified representation of the entire collection of fingerprints. The main utility of the flowFPPlex is its support for creating a merged representation of a set of instances acquired using a multitube panel, with different flowFPModels for each tube in the panel.

#### 2.2.4. Generic Functions

A number of other methods have been provided to facilitate interaction with and analysis of fingerprinting results. Chief among these are visualization methods that aid in the understanding and interpretation of fingerprinting results (see Figures S1–S3 in Supplementary Material available online at doi:10.1155/2009/193947). A few other accessor methods deserve special mention.

**nRecursions(obj)**. This generic function returns the number of levels of recursive subdivision of its argument. FlowFP, flowFPPlex, and flowFPModel all implement the method. Furthermore, the flowFP class implements the “set” method. This enables the user to compute a model at some fairly high resolution, and then to derive fingerprints at that resolution or any lower resolution without recomputing the model. This is possible because fingerprinting is recursive, so that given any high-resolution model, all models of lower resolution can be derived from it.

**counts(obj)**. This generic function returns a matrix of the number of events per instance and per bin. FlowFP and flowFPPlex classes implement this method, facilitating creation of fingerprint matrices suitable for processing by downstream methods outside of the flowFP package. The method has an optional argument “transformation” that can take on values “raw” (returns the actual event counts for each bin), “normalize” (normalizes by dividing raw counts by the expected number of events), or “log2norm” (like normalize except that it further takes the log_2_ of the result). 

**sampleNames(obj) and sampleClasses(obj)**. These generic functions set or get sample identifiers for objects of class flowFP or flowFPPlex. By default, for flowFPs, sample names are derived from the flowSet. However they can be overridden by the set method, providing flexibility to handle cases where the sample names in a flowSet are not appropriate. When adding fingerprints to a flowFPPlex, sample names (and if present, sample classes) are compared, and the join operation is not permitted unless names and classes among all fingerprints in the flowFPPlex are identical.

**parameters(obj)**. This generic function returns the light scatter and/or fluorescence variables involved in binning, either for a flowFPModel or a flowFP. The function is able to report both the variables that were considered for binning as well as those that actually participating (if the global variance of a variable is small enough it may never be selected for division).

**tags(fp)**. This generic function returns the tags slot of a flowFP object, described in Section 2.2.2. This is useful for visualization and gating operations.

**binBoundary(obj)**. This generic function reports a list of multivariate rectangles corresponding to the limits of the bins. FlowFP and flowFPModel classes both implement this method. This information is also useful for visualization and gating operations.

### 2.3. Data and Characteristics

Deidentified flow cytometric data from peripheral blood or bone marrow aspirate samples were provided by Clarient, Inc. (Aliso Viejo, CA) along with primary diagnoses by experienced hematopathologists. After application of QC filters including that described in Section 3.1.1 the data set included 42 cases diagnosed as Acute Myeloid Leukemia (AML) and 309 cases that were determined to be immunophenotypically normal. For the purposes of this study physician diagnosis was regarded as the ground truth.

Data were collected over a one-year period, using the panel described in Table 1. Briefly, samples were lysed with ammonium chloride, then washed with PBS, centrifuged and resuspended. Blocking was accomplished by incubating with RPMI-1640 supplemented with 10% rabbit serum for 30 minutes at 37°C. Cells were then pelleted, resuspended in RPMI-1640, and adjusted to between 4–8 × 10^6^ cells/mL. Antibody staining was accomplished by incubating in the dark at room temperature for 15 ± 5 minutes 100 *μ*L of the adjusted cell suspension with 40 *μ*L of pretitrated antibody cocktail per tube. For the viability tube, 10 *μ*L of 7AAD was added in place of the antibody cocktail. After staining each tube was washed with 3 mL PBS, vortexed, pelleted, and resuspended in 500 *μ*L of PBS prior to running on the flow cytometer. Five-color immunofluorescence along with forward and side scatter data were collected on two FC-500 cytometers (Beckman Coulter, Miami, FL). Data were collected for 3 × 10^4^ events for each tube.

## 3. Results

### 3.1. Gating Quality Control

#### 3.1.1. Tube Data

FlowFP was used to assess the consistency of event distributions in variables common to a multiple-tube panel. Using the panel described in Table 1, note that CD45 is common to all tubes except the viability tube. Frequently [16–22], the distribution of events in the Side Scatter versus CD45 projection (referenced as parameters 2 and 5 in the code below) from a single tube is used to gate an entire collection of tubes in order to save time. If the CD45 versus SSC distribution differs among the tubes, errors due to incorrect subsetting will occur, but may not be readily apparent without careful study of the gating plots.

Using flowFP, in order to rapidly detect consistency of CD45 versus SSC distributions without the need to look at dotplots, we (1) create a flowSet comprising tubes 1–7 of a sample, (2) create a model, using the common variables CD45 and SSC, from the flowSet, (3) create fingerprints of the same samples with respect to this model, and (4) display the result. The R commands to accomplish this using flowFP are as shown in Algorithm 1 (Code Fragment 1).

**Figure 1:**
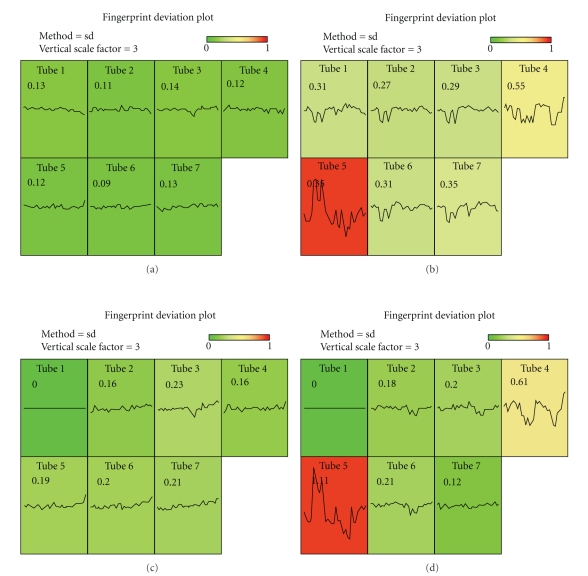
FlowFP plot method to display gating data consistency. Fingerprints were computed using CD45 and SSC which are common variables in all tubes. Fingerprint similarity is indicated by color and in the similarity metric shown in each panel. The color wedge shows mapping of colors to values of the similarity metric (values above the maximum indicated on the wedge all map to red). The *x*-axis for each subplot is fingerprint index, and the *y*-axis is the log_2_ transformed fingerprint value plotted with zero at the center and scaled to ± “vertical scale factor” (in this case 3.0). (a) Sample FI05_000942, an example of a sample with good gating consistency. (b) Sample FI05_000599, an example of a sample with poor gating consistency. (c) and (d) as in (a) and (b), except that models were computed from Tube 1 only, rather than the aggregate of Tubes 1–7 for each sample. Note that the fingerprint for Tube 1 in both cases has zero deviation, as expected. Note also the qualitative similarity between (a) and (c) and between (b) and (d).

Figure 1(a) shows the resulting plot. Each tube is represented by one of the colored plots, with the CD45 versus SSC fingerprint shown as a line. The standard deviation of the fingerprint values around their mean is shown for each tube to provide a quantitative measure of the degree to which a tube deviates from the norm of all tubes combined. The same value is mapped to colors, shown in the color legend above the plots, to provide a quick visual representation of the consistency of the distributions. For comparison, Figure 1(b) shows a similar result for a sample that displayed poor CD45 versus SSC consistency. Note that Tube 5 in that sample differed markedly from the other tubes in the panel, as did Tube 4, but to a lesser extent.

#### 3.1.2. 96-Well Plate Data

**Figure 2:**
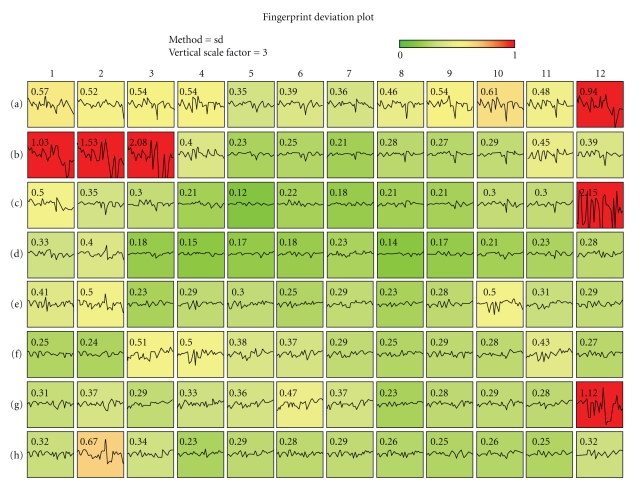
QC plot method for high-throughput data. Data were fingerprinted on variables common to all wells in a 96-well plate. The display maps into colors the degree to which gating data conform to the plate-wide norm.

High-throughput FC data are flexibly accommodated in the FlowFP package. For data derived from 96-well plates, a plot method of type “plate” can be used to display a qc-style plot in a layout that reflects the structure of the plate. Figure 2 shows such a result. Data were obtained [23, 24] in which SSC, CD3, and CD4 (parameters 2, 5, and 7) were used to gate the entire plate of data. The R commands shown in Algorithm 2 (Code Fragment 2) were used to produce the plot in Figure 2.

Note that in this case we illustrate the use of an implicit model by omitting the model from the flowFP constructor. The utility of such a rapid and straightforward quality assurance tool is most apparent in the case of this sort of high-throughput data.

### 3.2. Automated Classification of Acute Myeloid Leukemia

We now turn to the application of flowFP to support a machine learning workflow. The aim here is to illustrate the utility of fingerprint-based approaches in general, and flowFP in particular, by automatically categorizing samples into one of two *a priori* known classes, AML or Normal. The dataset described in Section 2.3 was used. Tube 1 (isotype control) and Tube 8 (viability) were ignored for the purpose of this analysis, leaving 6 tubes, numbered 2–7. 

We divided the samples randomly into a balanced training set comprising 21 of 42 AML cases and 21 of 309 Normal cases. We elected to balance the training set so as not to bias the classifier towards the more heavily represented Normal case. The remaining 21 AML cases and 288 Normal cases were assigned to the test set. Modeling and fingerprinting were done on a per-tube basis. Models were computed from training data only, in order to avoid biasing the prediction of the test set. We also employed a “differential modeling” procedure by creating two separate models, one for the AML training instances and one for the Normal training instances. Then, fingerprints from each tube and for each model were computed and aggregated into a flowFPPlex for further analysis. Fingerprinting was performed on all variables. The R code fragment implementing this procedure is shown in Algorithm 3 (Code Fragment 3).

Models were computed at a resolution level *l* = 11, producing *n* = 2048 bins. This resolution was determined using the default automatic setting of flowFPModel which implements the heuristic that the typical (median) number of events in each instance of the training set is binned such that the number of events per bin is not less than 8. The resulting flowFPPlex therefore had 6 tubes × 2 models × 2048 bins = 24 576 features.

We extracted feature values from the flowFPPlex using the accessor function counts(plex, transformation=“log2norm”) which performs a logarithmic transformation on the normalized counts matrix.

**Figure 3:**
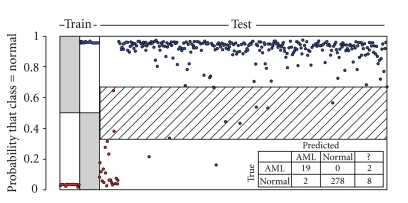
Support Vector Machine classification of AML versus Normal. The classifier was trained with 21 AML and 21 Normal Instances (left-most two regions). The classifier was then used to blindly predict class probabilities for the test set of 21 AML and 288 Normal instances (the right-most region). Ground-truth class assignments are indicated by color, red for AML and blue for Normal. The probability range 0-1 was divided into three equal regions. Instances falling into the lower third were classified as AML, in the upper third as Normal, and in the middle as Uncertain “?”.

Using only the instances in the training set, we performed a Mann-Whitney test on each feature independently (there are many methods of feature selection, a discussion of which is beyond the scope of this report). We selected those features which had a 99.9% likelihood of being differentially distributed between the two classes, after performing the Benjamini-Hochberg correction for multiple comparisons [25, 26]. This led to the selection of 1681 informative features out the original 24 576 features. Using the reduced feature set we trained a Support Vector Machine (SVM) classifier [27, 28] using a radial basis function kernel. We then blindly predicted the class of the test set using this classifier by assigning the predicted class probabilities into three equal ranges. The results are shown in Figure 3. Sensitivity and specificity are 90.5% (19/21) and 99.3% (278/288), respectively, with 9.5% (2) of AML instances and 2.8% (8) of Normal instances falling into the Uncertain group. No cross validation was performed here for clarity and brevity of presentation. For a better assessment of model performance this would be required. Interestingly, repeating the analysis without the “differential modeling” method described above (i.e., using AML and Normal combined training instances to compute the models for each tube) resulted in a similar result, but with a slightly poorer sensitivity of 85.7% (data not shown).

The time required to compute the fingerprints was 1.8 seconds per sample, requiring 5.2 GB of memory on a machine running the Linux 2.6 SMP 64-bit kernel with a 2.83 GHz processor. Recall that this represents, for each sample, the construction of 2 fingerprints for each of 6 tubes, each of which has 3 × 10^4^ events. Compared with mixture modeling approaches that are used for analysis of FC data (e.g., [8, 29]) flowFP is a computationally inexpensive method of analysis of FC data.

**Figure 4:**
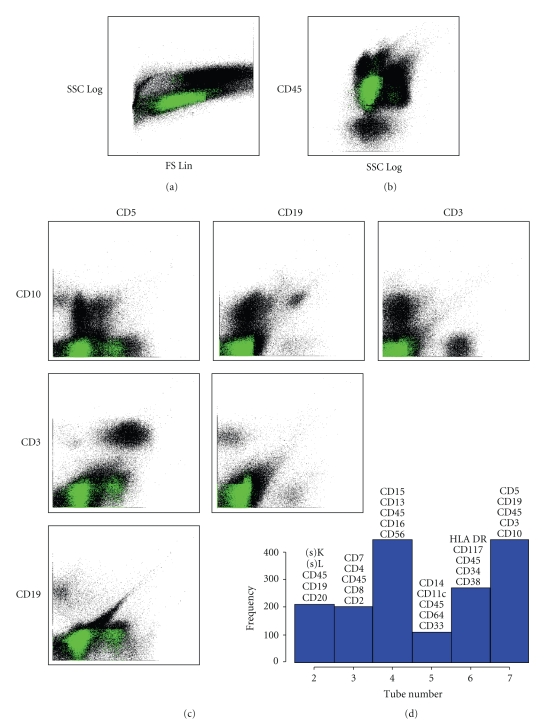
Visualization of informative features. (a)–(c) dotplots for Tube 7. Black dots are aggregated data from 5 AML and 5 Normal instances. Colored dots indicate events in informative bins with higher probability density in AML compared with Normal. (a) Side Scatter versus Forward Scatter. (b) CD45 versus Side Scatter. (c) Pairwise dotplots of fluorescence's CD5, CD19, CD3, and CD10. (d) Histogram of the frequency with which informative features occur in Tubes 2–7.

Figure 4(d) shows the distribution of informative features selected as described above with respect to tube number. Tubes 7 and 4 appear to be the most informative for distinguishing AML from Normal. Figures 4(a)–4(c)  display the informative subset of features (bins) that fell in Tube 7 and which had higher likelihood, on average, in the AML group compared with the Normal group. Informative features characteristic of AML can be described as low-intermediate SSC, CD45 dim, and negative for CD3, CD19, and CD10. The CD45 versus SSC distribution of the informative bins corresponds to a region containing blasts and monocytes.

**Figure 5:**
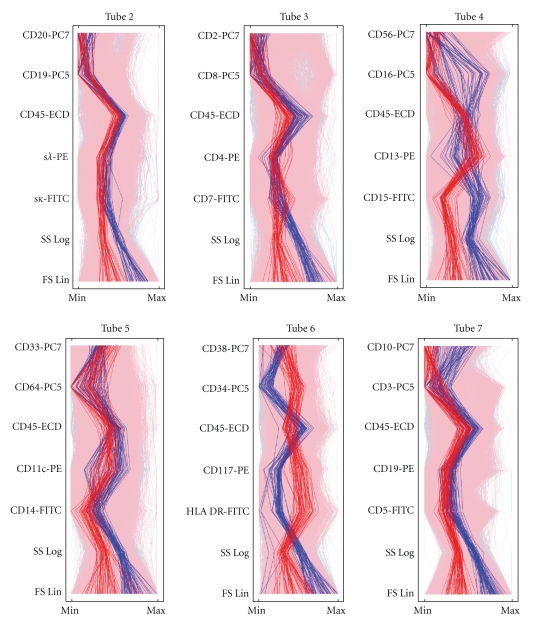
Parallel coordinate view of informative fingerprint features. The expression pattern of an individual event is shown as a vertical trajectory. Events are chosen from informative bins selected as described in the text. Events in bins with excess median probability among AML (Normal) instances are shown in red (blue). The numbers of AML and Normal trajectories are balanced to avoid visual bias.

A more comprehensive although less detailed picture of information distribution in the panel is illustrated in Figure 5. This parallel coordinate view enables the appreciation of expression patterns across the entire panel of tubes. Notice that the AML pattern in Tube 7 displayed in Figure 5 indicates the same CD45(dim), CD3(−), CD10(−) blast phenotype shown in Figure 4. In Tube 4 the phenotype of AML-informative bins is consistent with blasts expressing CD15(dim to −), CD13(dim to +), CD16(−), CD56(−) (see also Figure S4 in Supplementary Material). Separation of the bundles of trajectories corresponding to AML and Normal events is the widest in Tubes 4, 6, and 7, consistent with the distribution of information across the tubes shown in Figure 4(a). By contrast, Tube 5 has intertwined bundles, apparently in keeping with the fact that Tube 5 held the fewest informative fingerprinting features.

## 4. Discussion

With recent technological advances, FC is now capable of operating as a true high-throughput technique. A key enabling requirement however is the need to automate data analysis for speed, much as automation in sample preparation and data acquisition have accelerated the rate of generation of data and thereby enabled high-throughput FC. This requirement inevitably drives movement away from human-drawn, visually-based gating which is the single most significant obstacle preventing a true high-throughput FC workflow.

We have shown that fingerprint-based analysis of FC data represents an effective bridge between large amounts of FC data and the world of machine learning and knowledge discovery techniques. It effectively captures informative features of a multivariate probability distribution function and does so in a computationally efficient way. As such it represents one of the tools that may help to bring FC into a new era of application to problems previously not feasible due to limitations in data analysis techniques.

It is important to note that fingerprinting of FC data is not without limitations. First, we note that fingerprinting approaches are sensitive to differences in multivariate probability distributions no matter their origin. Thus, instrumental, reagent or other systematic variations may cause spurious signals as large or larger than true biological effects. For this reason it is important to measure and control for these effects [1]. In fact, fingerprinting itself can be used to assess and to help control for systematic effects, as was illustrated in Section 3.1. 

Second, because fingerprinting is, in essence, the creation of a multivariate histogram, it responds to factors that might artificially emphasize certain bins in preference to others. In particular, events may pile up on either the zero or full-scale axis for one or more variables. This situation frequently results from values that would be negative due to compensation or background subtraction (causing pileup on the zero axis) or at the other end of the scale, values that exceed the dynamic range of the signal detection apparatus causing pileup at full scale. At either end this results falsely in an apparent high density of events. Fingerprinting bins are thus “attracted” to these locations, causing a distortion in the proper characterization of the true multivariate probability distribution function. One might be tempted to simply remove these values. However this is problematic since they can be very important. For example, values piling up at full scale are the brightest of all. A better solution is to adjust detector gains to minimize or eliminate full-scale pileup, to use high-dynamic-range detectors and electronics and to use modified data transformations such as the biexponential transform to smoothly distribute values at or below zero.

Just as scaling and transformation of data are important for visualization of multivariable distributions [30–32], so they are also important for fingerprinting. Data acquired using linear amplifiers such as exist in some modern instruments, or data that have been “linearized” from instruments with logarithmic amplifiers, tend to be heavily skewed to the left, since in most cases data distributions are quasi-log-normally distributed. Bins determined from such data thus have extreme variations in size. A good rule of thumb is to use a data transformation that produces the most spread-out distribution, which also is often the transformation most effective for clear visualization of the distribution. For example, Forward Scatter data are almost always displayed on a linear scale, whereas fluorescence data are usually displayed on a logarithmic or biexponential scale. For a good review of scaling and transformation of flow cytometric data, the reader is referred to [32].

A key limitation for fingerprinting approaches, including flowFP, relates to the number of events available for analysis. Since the objective of probability binning is to find bins containing equal numbers of events, it follows that once the number of bins is on the order of the number of events in an instance, the expected number of events per bin will be of order unity. In this case differences in bin counts will not be statistically significant. On the other hand, if the dimensionality of the data set is high, the average number of times any variable will be divided in the binning process will be small. For example, in a dataset with 18 variables, if we demand at least, say, 10 events per bin for statistical accuracy, about 2.6 × 10^6^ events would be required in order that each variable is divided on average into at least two bins. Thus, the spatial resolution of binning is limited by the number of events collected, and as the number of variables increases, the number of events needed to maintain resolution increases geometrically.

FlowFP has been peer reviewed and accepted for inclusion in the next release of Bioconductor scheduled for October 2009. Prior to that date the development version may be downloaded from http://www.bioconductor.org/. The package is currently available for all architectures supported by Bioconductor. In addition to the functionality illustrated here, the authors plan to improve some of the visualization methods, specifically to enable better use of color, for example to represent statistical significance of bins. One of the advantages of integration with other flow cytometry Bioconductor packages is the ease of comparing and combining analysis methodologies. For example, it will be of interest to compare the performance of fingerprinting with other methods such as clustering and mixture modeling (flowClust). By the same token, such methods might be used in concert. For example, it is possible that clustering could be used to define major cell categories (e.g., B cells, T cells, granulocytes, etc.), within which fingerprinting may efficiently parse subsets correlated with function.

In summary, flowFP provides the flow cytometry community with a new tool that transforms FC data such that a wide range of other data analysis algorithms may be brought to bear. It creates a representation of FC data that preserves information embedded in the multivariate probability distribution function while at the same time presenting the information in a way that can be utilized easily by other software tools. Because it is tightly integrated in Bioconductor with several other FC-related packages and also exists in the broader R statistical computing environment, flowFP can interoperate with a very wide range of open-source analysis techniques. This power and flexibility enables a broad range of new computational analysis approaches that have potential in two distinct areas. First, it will facilitate the retrospective mining of FC data, seeking novel biomarkers that may be lurking in existing data. Second, it breaks the data analysis bottleneck that has up until now limited the full exploitation of FC in clinical applications.

## Supplementary Material

These supplementary figures illustrate some of the additional visualization capabilities of flowFP. Figure S1 shows the visualization of a flowFPModel. Figure S1(B) shows the bins of a 2-dimensional model. Figures S1(C-F) show the use of transparency in the display of bins to reveal the underlying distribution depicted in dot-plot form. Figure S2 shows several methods of illustrating fingperprints in order to facilitate comparisons across instances. Figure S3 links fingerprint index with the spatial location of events in specific fingerprint bins by way of color. Finally, Figure S4 shows the visualization of informative features derived from Tube 4, as described in Section 3.2 of the text.

## Figures and Tables

**Algorithm 1:**
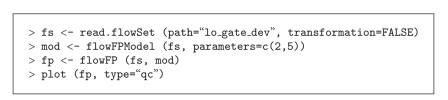
(Code Fragment 1).

**Algorithm 2:**
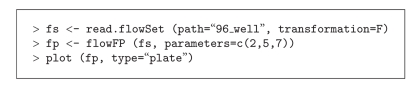
(Code Fragment 2).

**Algorithm 3:**
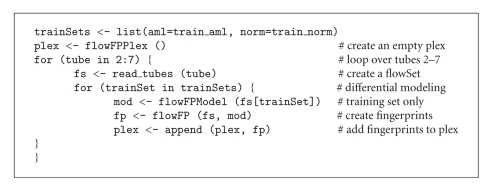
(Code Fragment 3).

**Table 1:** Reagent panel used for immunophenotyping of leukemia/lymphoma samples.

Tube	FL1	FL2	FL3	FL4	FL5
	P3S	P4S	P5S	P6S	P7S
1	IgG1-FITC	IgG1-PE	CD45-ECD	IgG1-PC5	IgG1-PC7
2	(s)Kappa-FITC	(s)Lambda-PE	CD45-ECD	CD19-PC5	CD20-PC7
3	CD7-FITC	CD4-PE	CD45-ECD	CD8-PC5	CD2-PC7
4	CD15-FITC	CD13-PE	CD45-ECD	CD16-PC5	CD56-PC7
5	CD14-FITC	CD11c-PE	CD45-ECD	CD64-PC5	CD33-PC7
6	HLA DR-FITC	CD117-PE	CD45-ECD	CD34-PC5	CD38-PC7
7	CD5-FITC	CD19-PE	CD45-ECD	CD3-PC5	CD10-PC7
8	FL1-Log	FL2-Log	FL3-Log	FL4-Log	FL5 Log
