# Supplementary material for: FlowFP: A Bioconductor Package for Fingerprinting Flow Cytometric Data
Source: Adv Bioinformatics. 2009 Sep 24;2009:193947. doi: 10.1155/2009/193947 (PMC2777013; doi:10.1155/2009/193947)
Supplement: Supplementary file 1 — These supplementary figures illustrate some of the additional visualization capabilities of flowFP. Figure S1 shows the visualization of a flowFPModel. Figure S1(B) shows the bins of a 2-dimensional model. Figures S1(C-F) show the use of transparency in the display of bins to reveal the underlying distribution depicted in dot-plot form. Figure S2 shows several methods of illustrating fingperprints in order to facilitate comparisons across instances. Figure S3 links fingerprint index with the spatial location of events in specific fingerprint bins by way of color. Finally, Figure S4 shows the visualization of informative features derived from Tube 4, as described in Section 3.2 of the text. [file 193947.f1.doc]

# flowFP: A Bioconductor Package for Fingerprinting Flow Cytometric Data

Wade T. Rogers and Herb A. Holyst

Department of Pathology and Laboratory Medicine, University of Pennsylvania School of Medicine, Philadelphia, PA.

Supplementary Materials


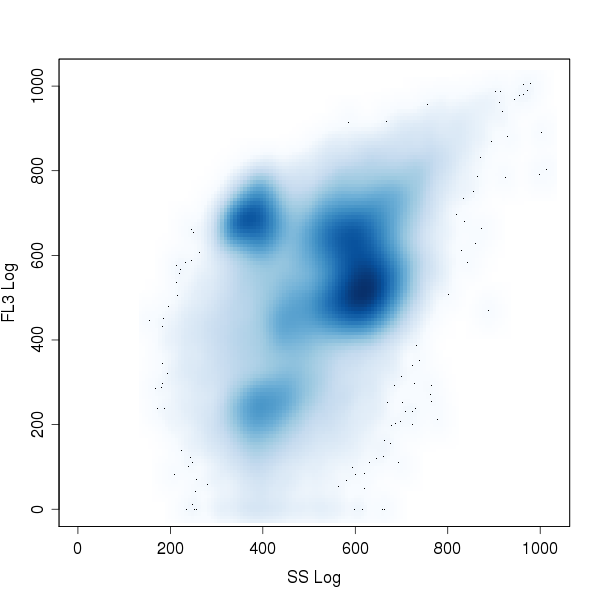

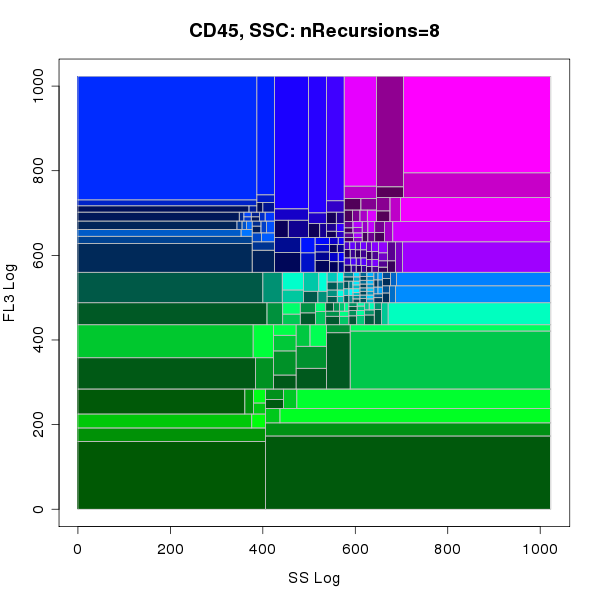

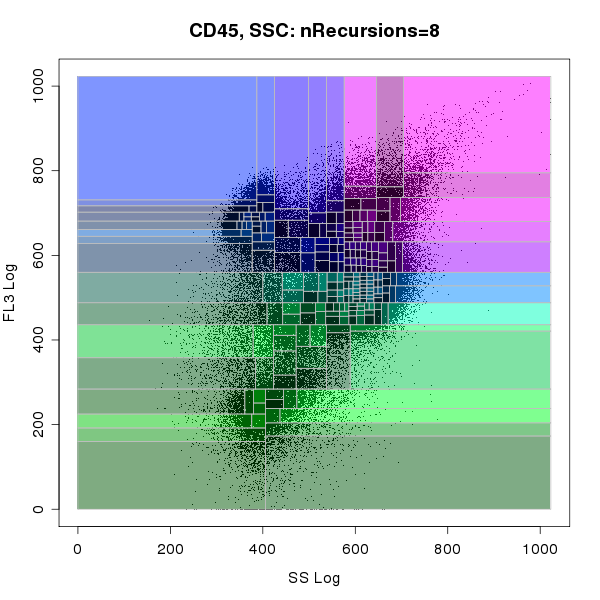

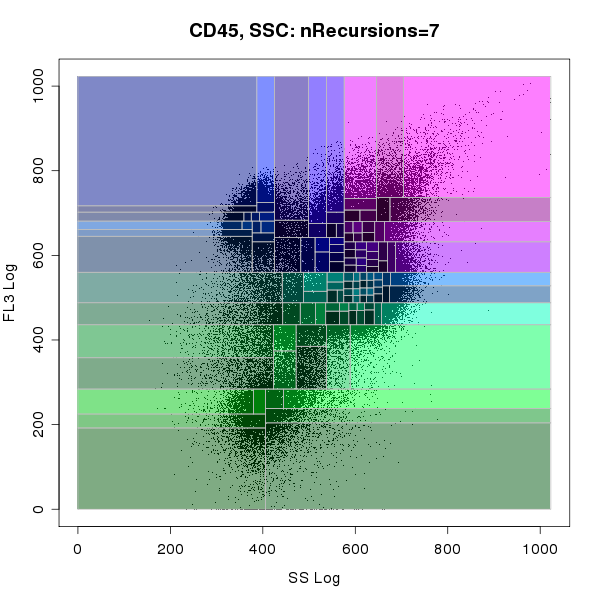

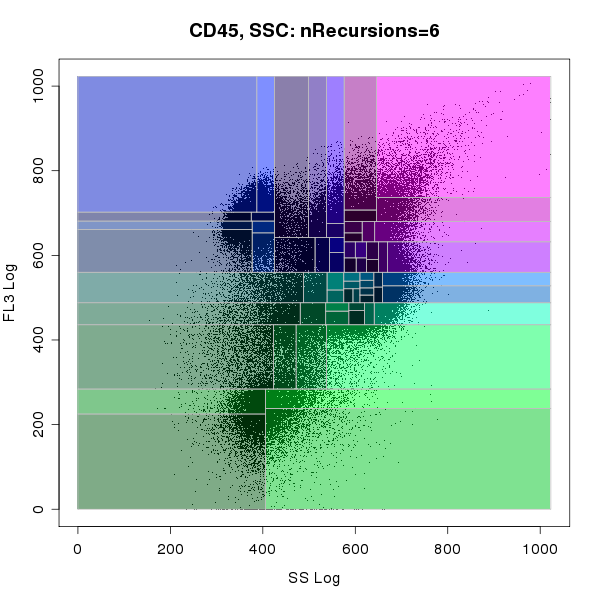

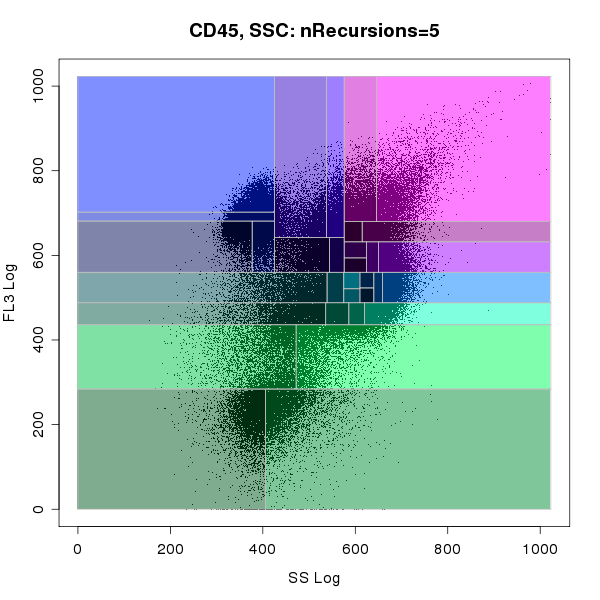


A

B

C

D

E

F

Supplementary Figure S1. Plot methods for a flowFPModel. (A) Dataset used for this illustration, shown on dimensions CD45 and SSC, used for fingerprinting in (B-F). (B) FlowFPModel of data in (A), using
plot(model). Note that bins are smaller in regions of higher event density. (C-F) Visualization of the model with the underlying flowSet shown as a dotplot, using plot(model, fs). The number of recursive subdivisions decreases from C to F, showing the resulting reduction in the sizes of the bins in the models.


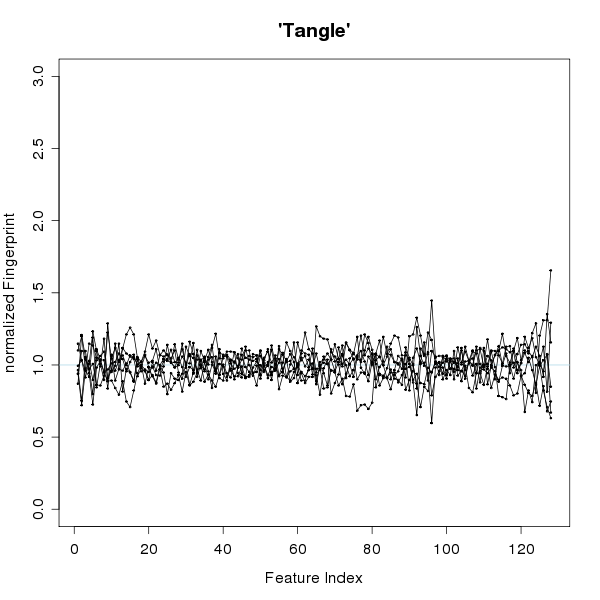

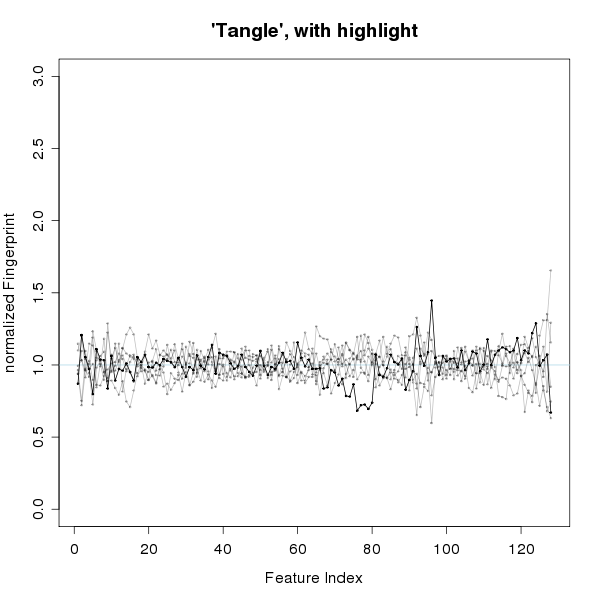

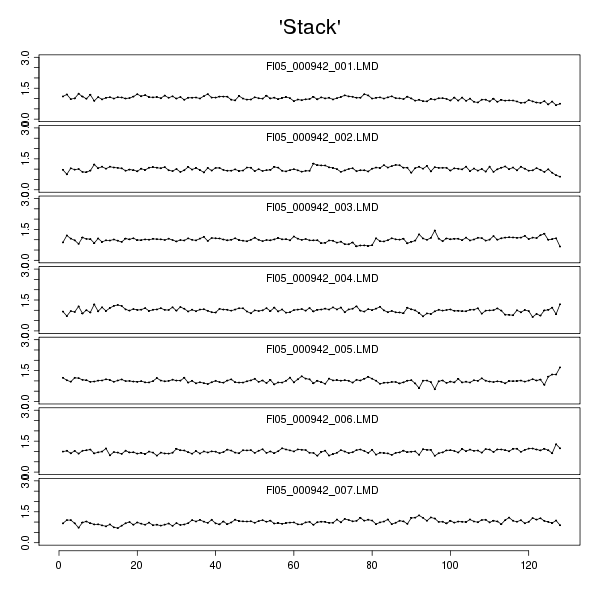

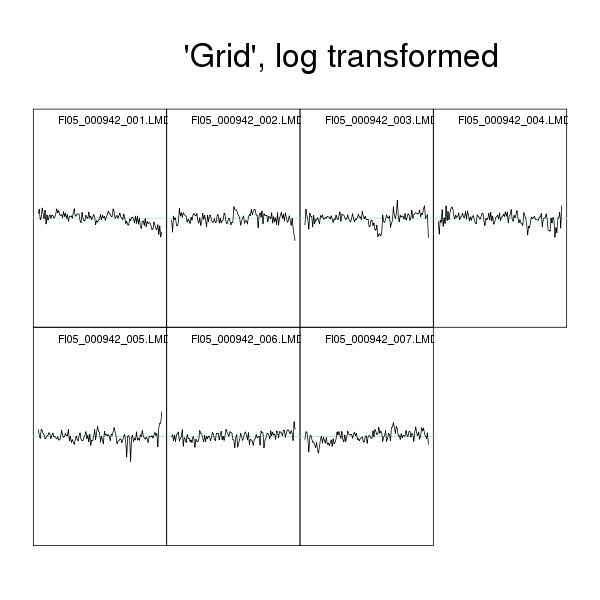


A

B

C

D

Supplementary Figure S2. Visualization methods for fingerprints. (A) ‘Tangle’ method, with fingerprints superimposed. (B) ‘Tangle’ method, showing highlighting of one of the seven fingerprints in the set. (C) ‘Stack” method. (D) ‘Grid’ method.


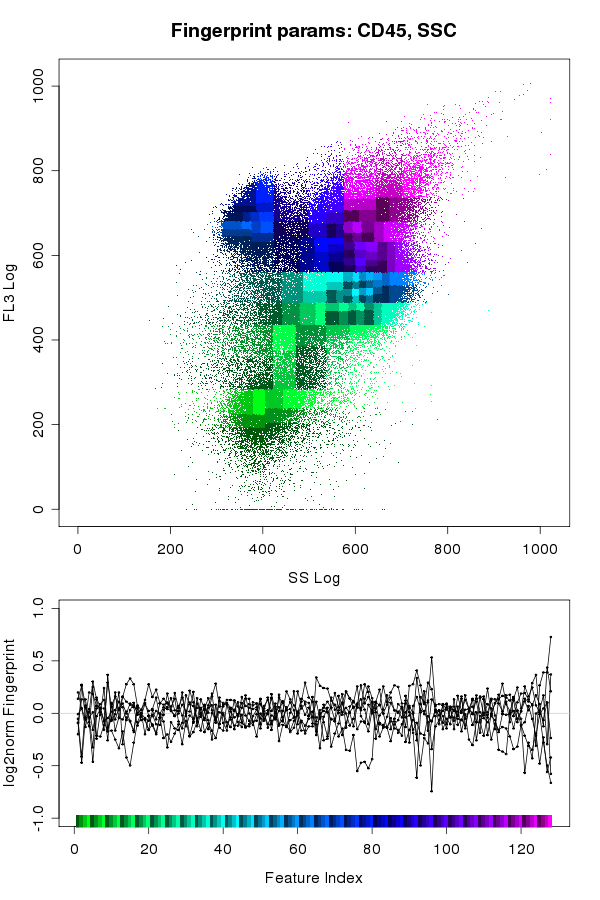


Supplementary Figure S3. Visualization method for fingerprints, showing the spatial relationship between fingerprint features (shown in the lower panel) and corresponding events (shown in the upper panel). Bin colors are shown at the bottom of the lower panel. For example, green hues correspond to lower feature indices, and violet colors to higher feature indices.


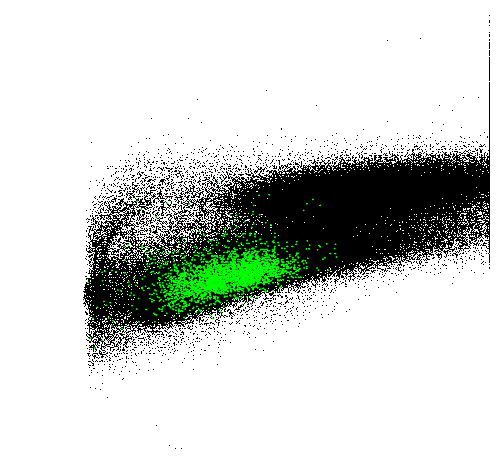

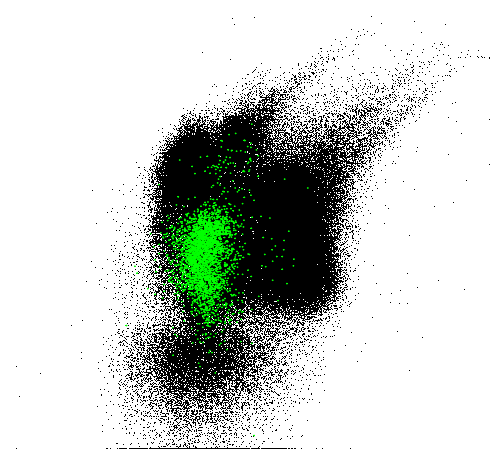

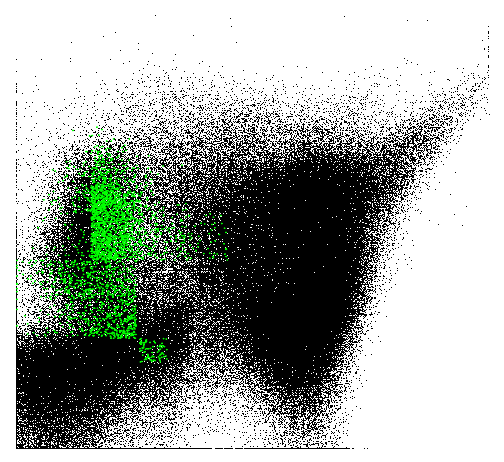

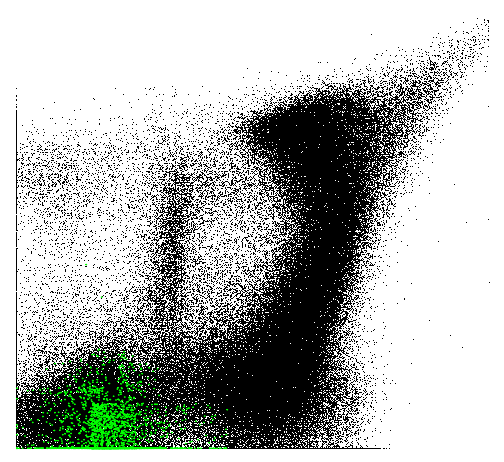

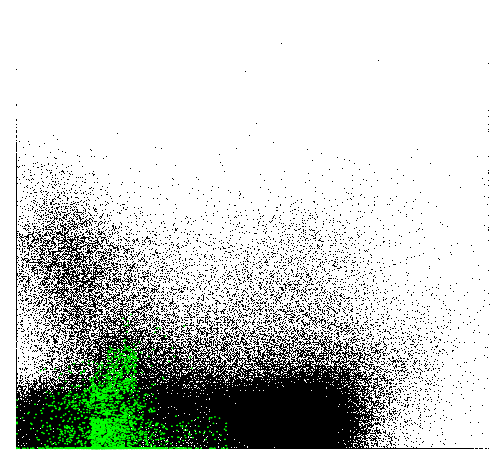

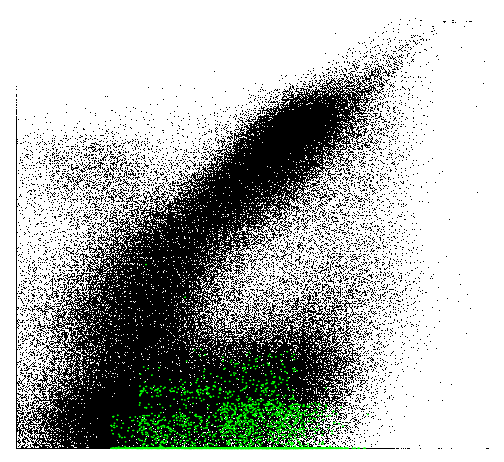

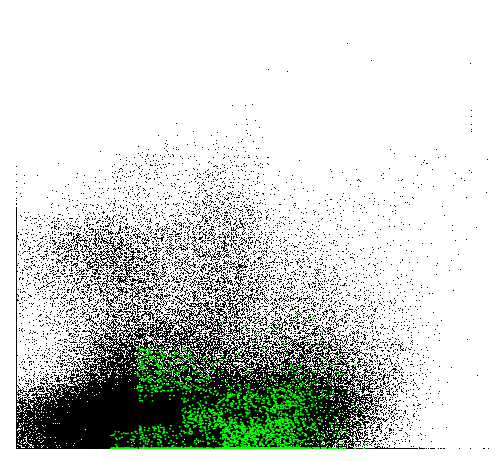

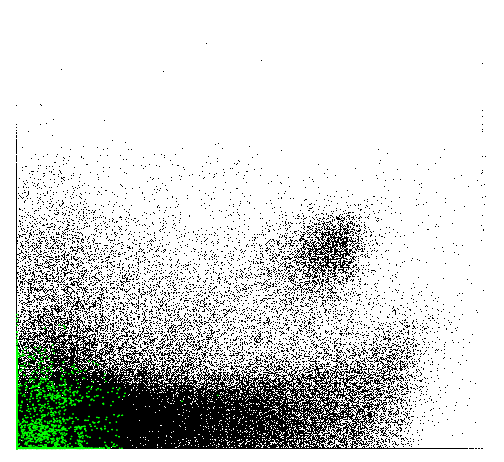


**CD56**

**CD16**

**CD13**

**CD15**

**CD13**

**CD16**

(C)

**FS Lin**

**SSC Log**

**SSC Log**

**CD45**

(A)

(B)

Supplementary Figure S4. Visualization of informative features for Tube 4. Black dots are aggregated data from 5 AML and 5 Normal instances. Colored dots indicate events in informative bins with higher probability density in AML compared with Normal. (A) Side Scatter vs. Forward Scatter. (B) CD45 vs Side Scatter. (C) Pairwise dotplots of fluorescence parameters CD15, CD13, CD16 and CD56.
